# Supplementary material for: Malaria transmission in Africa: Its relationship with yellow fever and measles
Source: PLoS One. 2022 May 4;17(5):e0268080. doi: 10.1371/journal.pone.0268080 (PMC9067666; doi:10.1371/journal.pone.0268080)
Supplement: Attachment — Submitted filename: PONE-D-21-27941_R1 (1).pdf [file pone.0268080.s003.pdf]

PLOS ONE

Malaria transmission in Africa: its relationship with yellow fever and measles

--Manuscript Draft--

**Manuscript Number:**	PONE-D-21-27941R1
**Article Type:**	Research Article
**Full Title:**	Malaria transmission in Africa: its relationship with yellow fever and measles
**Short Title:**	Malaria and vaccine preventable diseases
**Corresponding Author:**	Oyetunde Oyeyemi<br>UNIMED: University of Medical Sciences Ondo City<br>Ondo, Ondo NIGERIA
**Keywords:**	Malaria; Vaccine-preventable diseases; modelling; relationships; Africa
**Abstract:**	Background<br>Malaria has been strongly linked to the transmission and pathophysiology of some viral diseases. Few studies are however available on the possible relationships between malaria and vaccine-preventable diseases. The study aims to evaluate the relationships between the incidence of malaria and cases of measles and yellow fever in Africa.<br>Methods<br>The malaria incidence, death due to malaria, measles and yellow fever data were sourced from the WHO database. Poisson and zero-inflated time-trend regression were used to model the relationships between malaria and the two vaccine-preventable diseases.<br>Results<br>A significant negative relationship existed between malaria incidence and measles cases (P<0.05), however, malaria showed a positive relationship with yellow fever (P<0.05). The relationships between death due to malaria and measles/yellow fever cases followed similar trends but with a higher level of statistical significance (P<0.001).<br>Conclusions<br>The relationships observed in this study could be important for the management of malaria and the studied vaccine-preventable diseases. Increase vaccination coverage and/or malaria treatment could modulate the direction of these relationships.
**Order of Authors:**	Oluyemi Okunlola
	Oyetunde Oyeyemi
**Response to Reviewers:**	Response to comments<br><br>We thank you for your efforts both in giving our manuscript quality review and expediting the process. As suggested and requested by the Editor and the Reviewer, we have provided responses to the concerns raised. Our responses are appended below:<br><br>Editor's comments<br>1. We have now adapted the manuscript as suggested to the journal's style<br>2. Online profile is now update to include the ORCID number of the corresponding<br>3. We have now removed the phrase "data not shown" and associated sentence from the manuscript since the data are not a core part of the research being presented in our study.<br>4. Figures 1 and 2 are original and we generated by the software QGIS (version 3.14). This was stated in the statistical analyses. So, they have no copyright issues.<br>5. Data availability statement has been revised and the website included. The file containing the minimal data is also included as supplementary file.<br><br>Reviewer #1: General comments<br>This is an important subject in Africa, the continent with the highest burden of malaria, but of recently experiencing outbreaks due to viral diseases.<br>Line 69: Authors should change the word "Precious" to "Previous"

	Response – Thank you for the observation. It has now been corrected<br>Line 207. The statement is not correct. Rephrase to read "Fever in each of African Region and Africa as a whole"<br>Response – Thank you. This is now corrected as requested<br>Line 236: The word ITN has been introduced without being defined anywhere in the manuscript<br>Response – Thank you for the observation, it is now defined at first mention (please see line 224)<br>Lines 236-237: The statement must be supported by appropriate References<br>Response – Thank you, we have now included references<br>Line 276: The mention of Culex mosquito is irrelevant and confusing. The mosquito species neither transmit malaria no Yellow fever.<br>Response – Thank you, we have deleted Culex mosquitoes as advised
**Additional Information:**	
**Question**	**Response**
**Financial Disclosure**<br><br>Enter a financial disclosure statement that describes the sources of funding for the work included in this submission. Review the submission guidelines for detailed requirements. View published research articles from *PLOS ONE* for specific examples.<br><br>This statement is required for submission and **will appear in the published article** if the submission is accepted. Please make sure it is accurate.<br><br>**Unfunded studies**<br>Enter: *The author(s) received no specific funding for this work.*<br><br>**Funded studies**<br>Enter a statement with the following details:<br>• Initials of the authors who received each award<br>• Grant numbers awarded to each author<br>• The full name of each funder<br>• URL of each funder website<br>• Did the sponsors or funders play any role in the study design, data collection and analysis, decision to publish, or preparation of the manuscript?<br>• **NO** - Include this sentence at the end of your statement: *The funders had no role in study design, data collection and analysis, decision to publish, or preparation of the manuscript.*<br>• **YES** - Specify the role(s) played.<br><br>* typeset	The author(s) received no specific funding for this work

**Competing Interests**<br><br>Use the instructions below to enter a competing interest statement for this submission. On behalf of all authors, disclose any competing interests that could be perceived to bias this work—acknowledging all financial support and any other relevant financial or non-financial competing interests.<br><br>This statement is required for submission and **will appear in the published article** if the submission is accepted. Please make sure it is accurate and that any funding sources listed in your Funding Information later in the submission form are also declared in your Financial Disclosure statement.<br><br>View published research articles from *PLOS ONE* for specific examples.<br><br>**NO authors have competing interests**<br><br>Enter: *The authors have declared that no competing interests exist.*<br><br>**Authors with competing interests**<br><br>Enter competing interest details beginning with this statement:<br><br>*I have read the journal's policy and the authors of this manuscript have the following competing interests: [insert competing interests here]*<br><br>* typeset	The authors have declared that no competing interests exist
**Ethics Statement**<br><br>Enter an ethics statement for this submission. This statement is required if the study involved:<br><br>• Human participants<br>• Human specimens or tissue<br>• Vertebrate animals or cephalopods	N/A

• Vertebrate embryos or tissues<br>• Field research<br><br>Write "N/A" if the submission does not require an ethics statement.<br><br>General guidance is provided below. Consult the submission guidelines for detailed instructions. **Make sure that all information entered here is included in the Methods section of the manuscript.**	

Powered by Editorial Manager® and ProduXion Manager® from Aries Systems Corporation

**Format for specific study types**<br><br>**Human Subject Research (involving human participants and/or tissue)**<br>• Give the name of the institutional review board or ethics committee that approved the study<br>• Include the approval number and/or a statement indicating approval of this research<br>• Indicate the form of consent obtained (written/oral) or the reason that consent was not obtained (e.g. the data were analyzed anonymously)<br><br>**Animal Research (involving vertebrate animals, embryos or tissues)**<br>• Provide the name of the Institutional Animal Care and Use Committee (IACUC) or other relevant ethics board that reviewed the study protocol, and indicate whether they approved this research or granted a formal waiver of ethical approval<br>• Include an approval number if one was obtained<br>• If the study involved *non-human primates*, add *additional details* about animal welfare and steps taken to ameliorate suffering<br>• If anesthesia, euthanasia, or any kind of animal sacrifice is part of the study, include briefly which substances and/or methods were applied<br><br>**Field Research**<br><br>Include the following details if this study involves the collection of plant, animal, or other materials from a natural setting:<br>• Field permit number<br>• Name of the institution or relevant body that granted permission	
**Data Availability**<br><br>Authors are required to make all data underlying the findings described fully available, without restriction, and from the time of publication. PLOS allows rare exceptions to address legal and ethical concerns. See the PLOS Data Policy and FAQ for detailed information.	Yes - all data are fully available without restriction

A Data Availability Statement describing where the data can be found is required at submission. Your answers to this question constitute the Data Availability Statement and **will be published in the article**, if accepted.<br><br>**Important:** Stating 'data available on request from the author' is not sufficient. If your data are only available upon request, select 'No' for the first question and explain your exceptional situation in the text box.<br><br>Do the authors confirm that all data underlying the findings described in their manuscript are fully available without restriction?	
**Describe where the data may be found in full sentences. If you are copying our sample text, replace any instances of XXX with the appropriate details.**<br><br>• If the data are **held or will be held in a public repository**, include URLs, accession numbers or DOIs. If this information will only be available after acceptance, indicate this by ticking the box below. For example: *All XXX files are available from the XXX database (accession number(s) XXX, XXX.).*<br>• If the data are all contained **within the manuscript and/or Supporting Information files**, enter the following: *All relevant data are within the manuscript and its Supporting Information files.*<br>• If neither of these applies but you are able to provide **details of access elsewhere**, with or without limitations, please do so. For example:<br><br>*Data cannot be shared publicly because of [XXX]. Data are available from the XXX Institutional Data Access / Ethics Committee (contact via XXX) for researchers who meet the criteria for access to confidential data.*<br><br>*The data underlying the results presented in the study are available from (include the name of the third party*	The variables data examined in the study are available from the WHO database (www.who.int/data/gho)

and contact information or URL).<br>• This text is appropriate if the data are owned by a third party and authors do not have permission to share the data.<br><br>* typeset	
Additional data availability information:	

Manuscript Click here to access/download;Manuscript;Manuscript.docx

Malaria transmission in Africa: its relationship with yellow fever and measles

Running head - Malaria and vaccine preventable diseases

Oluyemi A. Okunlola[1] and Oyetunde T. Oyeyemi[2*]

[1]Department of Mathematics, University of Medical Sciences, Ondo, Ondo State, Nigeria

[2]Department of Biological Sciences, University of Medical Sciences, Ondo, Ondo State, Nigeria

[*] Corresponding author: Oyetunde T. Oyeyemi

Address: Department of Biological Sciences, University of Medical Sciences, Ondo, Ondo State, Nigeria.

E-mail address: ooyeyemi@unimed.edu.ng

Phone number: +2348163546787

Conflict of interest statement for all authors: authors declared no conflict of interest

Access to data: All authors had access to the data and a role in writing the manuscript

Article type: Research Article

Key words; Malaria, Vaccine-preventable diseases, Modelling, Relationships, Africa

Abstract

Background

Malaria has been strongly linked to the transmission and pathophysiology of some viral diseases. Few studies are however available on the possible relationships between malaria and vaccine-preventable diseases. The study aims to evaluate the relationships between the incidence of malaria and cases of measles and yellow fever in Africa.

Methods

The malaria incidence, death due to malaria, measles and yellow fever data were sourced from the WHO database. Poisson and zero-inflated time-trend regression were used to model the relationships between malaria and the two vaccine-preventable diseases.

Results

A significant negative relationship existed between malaria incidence and measles cases ($P<0.05$), however, malaria showed a positive relationship with yellow fever ($P<0.05$). The relationships between death due to malaria and measles/yellow fever cases followed similar trends but with a higher level of statistical significance ($P<0.001$).

Conclusions

The relationships observed in this study could be important for the management of malaria and the studied vaccine-preventable diseases. Increase vaccination coverage and/or malaria treatment could modulate the direction of these relationships.

Key words: Malaria, Vaccine-preventable diseases, Modelling, Relationships, Africa

Introduction

Malaria is the most important parasitic disease in the tropical and sub-tropical regions of the world. In 2017, an estimated 219 million malaria cases and 435 000 malarial-associated deaths were recorded globally [1]. Africa is the most affected region of the world; accounting for more than 90% malaria cases and associated deaths [1]. The recommended malaria interventions include prompt detection of infection, treatment of clinical and severe malaria, vector control, and chemoprevention in high-risk groups [2].

Yellow fever is a vector-borne disease caused by the yellow fever virus of the genus *Flavivirus* [3]. With estimated 51 000 to 380 000 severe cases of yellow fever, and 19 000 to 180 000 deaths per year in Africa [4], the continent is the epicenter of the disease recording 90% of the total global cases of the disease [5]. Approximately half of the infected populations are asymptomatic and a further third suffer from a mild illness. Only about 12% of yellow fever infections present a severe disease in the form of jaundice and/or haemorrhage with 30-60% death rate [6]. Measles, like malaria and yellow fever, is another disease with serious public health implications for children. Although the global transmission of measles has been greatly reduced by vaccination about 60 years ago, it continues to be one of the major causes of mortality among children in sub-Saharan Africa [7].

Previous studies have related malaria with some viral infections, especially HIV and hepatitis [8-10], and these studies suggested the possible impact of malaria on the dynamics of transmission and morbidities associated with viral infections. Despite the dreadfulness of malaria and viral agents in the current study, studies on concomitant presentation of these diseases are very rare. Although few epidemiological studies are available for malaria and yellow fever [11,12], none is available for malaria and measles. Other studies involving the triads included the effect of

chloroquine prophylaxis on yellow fever vaccine antibody response [13], and impact of placental malaria on infant immunity to measles.

The aim of the current study is to determine the magnitude and direction of the trend of malaria incidence, death due to malaria, measles cases and yellow fever cases in selected African countries. Also to investigate the effect of measles and yellow fever cases on malaria incidence and death due to malaria, respectively. Because all the studied variables are cases and rates which are regarded as count data, a Panel Poisson regression model was adopted to understudy these relationships. Where there are observed excess zero in the cases of event considered in the study, a zero-inflated Poisson (ZIP) regression that is robust to the occurrence of zero is adopted to achieve our specified objectives. This study could have implications on the control of malaria and these vaccine-preventable diseases.

Material and methods

Data and scope

The variables data examined in the study were sourced from the WHO database (www.who.int/data/gho) from 2000-2018 for both malaria incidence and death due to malaria while the year 1980-2019 was used for measles and yellow fever cases for all African countries with a well-filled data on the mentioned variables. Consequently, 39 countries were included for malaria incidence, 28 countries for death due to malaria, 52 countries for measles and 30 countries for yellow fever annual trend change model.

Regression analyses

Count data are known to be discrete variables and not continuous, thus, the traditional regression model that assumes that outcome or response variable comes from a normal distribution is not

tenable bearing in mind that discrete variable is highly skewed. This study used a discrete probability distribution that treats the response variable of count observation as a Poisson process with probability mass function given as $f(y) = \frac{e^{-\mu}\mu^{y}}{y!}$, $0 \leq y \leq \infty$ (1)

This bound indicates that the distribution takes on nonnegative random numbers. The quantity λ is the mean of the Poisson distribution. This distribution has a unique property of equi-dispersion that the mean, $\lambda = E(y)$ is equal to variance, $V(y)$. However, the assumption of equi-dispersion is highly restrictive and hardly satisfied in real-life application as shown here that the mean each variable considered is not equal to the variance. Two major problems that are associated with Poisson regression are over-dispersion and under-dispersion which connote situations where $V(y) > E(y)$ and $V(y) < E(y)$, respectively. Sellers and Shmueli [14] provided a more general count distribution that captured a wide range of dispersion which they titled Conway–Maxwell-Poisson (COM-Poisson) and Huang [15] provided a COM-Poisson Double Generalized Linear Model that addressed the problem of both under and overdispersion simultaneous.

Poisson regression is achieved by expressing λ in equation (1) as a function of some explanatory variables through a log link function. This can be mathematically expressed as:

$\lambda_i = X'\beta$ or $\mu_i = \exp(X'\beta)$ (2)

Given the density function of equation (1) type and set of independent variable X_i, the maximum likelihood estimates of regression parameter β is defined as;

$log_e \, L(\beta) = \sum\{Y_i X'\beta - \exp(X'\beta) - \ln(Y!)\}$ (3)

Count data most often have excess zero cases and if not adjusted for can lead to inaccurate regression estimate and misleading conclusion. Lambert [16] developed a zero-inflated Poisson (ZIP) regression that corrects for the occurrence of excess zero cases in count data. The ZIP has

become a popular choice in empirical problems relating to count data. The ZIP model is often appealing, first, since it divides the dependent variable into two subpopulations where the first one takes on the value of zero with probability π_i while the second one is Poisson distributed with probability $1 - \pi_i$ [17]. Due to this setup, the ZIP model may be used when the data contain an excess amount of zeros. Second, this model is popular because it accounts for overdispersion (meaning that the variance of the dependent variable exceeds the mean value), which leads to an underestimation of the variance of the estimated coefficients when applying the standard Poisson regression model [17]. The ZIP divides the count data into two non-overlapping subpopulations using the equation:

$$y_i = \begin{cases} 0 & with\ porbability\ \pi_i \\ P_0(\mu_i) & with\ porbability\ 1 - \pi_i \end{cases} \text{ and } \pi_i = \frac{\exp(q_i'\gamma)}{1+\exp(q_i'\gamma)} \qquad (3)$$

In this expression q_i is the ith row of the Q, which is the data matrix for the logit model and γ corresponds to a $(p+1) \times 1$ vector of coefficients. Also, $\mu_i = \exp(x_i'\beta)$, where x_i is the ith row of X, which is the data matrix for the ZIP model while β corresponds to a $(p+1) \times 1$ vector of coefficients. The most common method of estimating β is to apply the ML method. By defining 1(*yi* = 0*)* as an indicator function that takes on the value of 1 if $y_i = 0$, the following joint likelihood should then be maximized:

$$L(\beta,\gamma) = \sum_{i=1}^{N}(l(y_i = 0)(\log(\exp(q_i'\gamma)) + \exp(-\exp(-\exp(X_i'\beta)))) + \sum_{i=1}^{N}(1 - l(y_i = 0)(y_i', x_i'\beta - \exp(x_i'\beta - \exp(x_i'\beta))] - \sum_{i=1}^{N}\log(1 + \exp(q_i'\gamma)) \qquad (4)$$

This complex likelihood function is estimated through the modification of the simplex method of Nelder and Mead [18] proposed by Kibria et al. [17] in which the start-up values come from the iterative weighted least-squares algorithm of the individual Poisson and logit estimation. The modification to this method applied the estimator specified in (5).

$$\hat{\beta}_{RR} = (X`\widehat{W}X + kl)^{-1}X`\widehat{W}X\hat{\beta}_{ML} \quad (5)$$

$\hat{\beta}_{ML}$is the estimate obtained using the simplex method. Furthermore, the shrinkage parameter k may take on values between zero and infinity, and when k equals zero, we have $\hat{\beta}_{RR} = \hat{\beta}_{ML}$. When k is greater than zero, we have $\|\hat{\beta}_{RR}\| < \|\hat{\beta}_{ML}\|$. Since $\hat{\beta}_{ML}$ is, on average, too long in the presence of multicollinearity, $\hat{\beta}_{RR}$ is expected to perform better than $\hat{\beta}_{ML}$.

Statistical analysis

The statistical analysis was carried out using five statistical software packages. SPSS version 23 was used for data management, cleaning and descriptive analysis. STATA version 13 and R version 4.0.3 were used to estimate the Poisson and zero-inflated Poisson (ZIP) regression model. GeoDA 1.9 was used to prepare the shapefile and merging of the estimated annual average change (EAAC) while QGIS version 3.14 was used for geospatial mapping of the EAAC of each variable.

Results

Time trend Poisson and zero-inflated Poisson model was fitted for malaria incidences (47 countries), death due to malaria (46 countries), measles cases (54 countries) and yellow fever cases (22 countries). Poisson regression was used for 46(97.87%), 46(100.0%) and 47(87.04%) for malaria incidence, death due to malaria and measles cases while zero-inflated Poisson was used in 1(2.13%), 7(12.06%) and 22(100.0%) countries where there were clear occurrences of excess zero in the malaria incidence, measles cases and yellow fever cases respectively. Malaria incidence reduced significantly in 38(80.9%) countries, increased significantly in 5(10.6%) and increased insignificantly in 4(8.51%) of out the 47 countries observed in the study

over time. Death due to malaria decreased significantly in 20(43.5%) countries but increased significantly in 24(52.2%) of the 46 countries included for the study over time. Also, of the total 54 countries studied for measles cases, 49(90.7%) showed significant decrease trend, 4(7.4%) significant increase trend and 1(1.85%) insignificant time trend coefficient. Yellow fever cases decreased significantly in 13(59.1%), increased significantly in 3(13.6%) countries while the remaining countries 6(27.27%) showed an insignificant trend (Table 1).

The spatial distribution of malaria incidence in African countries is presented in Fig 1. Malaria incidence average annual trend decreased significantly in several African countries except in Algeria, Cape Verde, Mali and Eswatini with time trend regressions -0.088, -0.016, -0.002 and -0.037, respectively, which showed a non-significant trend ($P>0.05$). A significant increase in malaria incidence was recorded in Djibouti (0.144), Eritrea (0.046), Madagascar (0.024), Rwanda (0.078), and Niger (0.008) ($P<0.05$) (Table 1).

Fig 1. Spatial Distribution of Malaria (A) and Death due to Malaria (B) in Africa.

The spatial distribution of malaria-associated death in African countries is presented in Fig 1. Algeria with trend regression (0.257) recorded the highest increase in death due to malaria while Egypt (-0.514), on the other hand, recorded the highest decrease in death due to malaria ($P<0.05$). Several countries in East Africa recorded a significant increase in malaria-associated death while many countries in Central Africa however, showed a significant decrease in malaria-associated death ($P<0.05$). All the countries in the South African region recorded a significant increase in death due to malaria ($P<0.05$) (Table 1).

The spatial distribution of measles in African countries is presented in Fig 2. Majority of the African countries recorded a significant decrease in measles cases except in Mauritius (0.042),

Somalia (0.063), South Sudan (0.058), and Democratic Republic of Congo (0.062) (P<0.05). Seychelles (-0.006) showed a non-significant decrease in measles cases annual trend (P>0.05) (Table 1). The spatial distribution of yellow fever cases is presented in Fig 2. Chad with trend regression 0.268 recorded the highest significant increase in yellow fever cases while Congo (-0.236) showed the highest significant decrease in yellow fever cases in Africa (P<0.05). The Central Africa Republic (-0.017), Burkina Faso (0.003), Côte d'Ivoire (0.004), Guinea-Bissau (0.03), Mauritania (-0.047), and Togo (0.039) recorded non-significant yellow fever cases trends (Table 1).

Fig 2. Spatial Distribution of Measles (A) and Yellow Fever Cases (B) in Africa.

Table 1. Poisson and Zero Inflated Time Trend Regression

		Malaria Incidence			Death due to Malaria			Measles Cases			Yellow fever Cases			
Region	Country	Method	Estimate	Significance	Method	Estimate	Significance	Method	Estimate	Significance	Method	Estimate	YFTD	Significance
North	Algeria	P	-0.088	No	P	0.257	Yes (5%)	P	-0.082	Yes				
North	Egypt				P	-0.514	Yes (5%)	P	-0.045	Yes				
North	Libya							P	-0.041	Yes				
North	Morocco	ZIP	-0.405	Yes	P	0.13	No	P	-0.17	Yes				
North	Sudan	P	-0.046	Yes	P	-0.144	Yes	P	-0.111	Yes				
North	Tunisia							P	-0.064	Yes				
East	Burundi	P	-0.05	Yes	P	-0.171	Yes	P	-0.135	Yes				
East	Comoros	P	-0.084	Yes				ZIP	-0.087	Yes				
East	Djibouti	P	0.144	Yes	P	-0.055	No	P	-0.117	Yes				
East	Eritrea	P	0.046	Yes	P	-0.018	Yes	P	-0.061	Yes				
East	Ethiopia	P	-0.074	Yes	P	0.093	Yes	P	-0.039	Yes				
East	Kenya	P	-0.08	Yes	P	0.165	Yes	P	-0.175	Yes	ZIP	-0.119	1	Yes
East	Madagascar	P	0.024	Yes	P	0.019	Yes	P	-0.042	Yes				
East	Malawi	P	-0.036	Yes				P	-0.126	Yes				
East	Mauritius							ZIP	0.042	Yes				
East	Mozambique	P	-0.025	Yes	P	0.125	Yes	P	-0.066	Yes				
East	Rwanda	P	0.078	Yes	P	0.168	Yes	P	-0.168	Yes				
East	Seychelles							ZIP	-0.006	No				
East	Somalia	P	-0.104	Yes	P	0.078	Yes	P	0.063	Yes				
East	South Sudan	P	-0.029	Yes	P	-0.255	Yes	P	0.058	Yes				
East	Uganda	P	-0.038	Yes	P	-0.011	Yes	P	-0.04	Yes	ZIP	-0.071	1	Yes
East	United Republic of Tanzania	P	-0.07	Yes	P	0.013	Yes	P	-0.139	Yes				
East	Zambia	P	-0.049	Yes	P	0.101	Yes	P	-0.128	Yes				
East	Zimbabwe	P	-0.033	Yes	P	0.145	Yes	ZIP	-0.077	Yes				
Central	Angola	P	-0.035	Yes	P	0.035	Yes	P	-0.084	Yes	ZIP	0.11	2	Yes
Central	Cameroon	P	-0.034	Yes	P	-0.026	Yes	P	-0.107	Yes	ZIP	-0.032	1	Yes
Central	Central African Republic	P	-0.016	Yes	P	-0.165	Yes	P	-0.065	Yes	ZIP	-0.017	1	No

Central	Congo	P	-0.033	Yes	P	-0.078	Yes	P	-0.116	Yes	ZIP	-0.236	1	Yes
Central	Democratic Republic of the Congo	P	-0.028	Yes	P	-0.075	Yes	P	0.062	Yes	ZIP	-0.136	1	Yes
Central	Equatorial Guinea	P	-0.02	Yes	P	-0.161	Yes	P	-0.101	Yes				
Central	Gabon	P	-0.007	Yes (5%)	P	0.192	Yes	P	-0.085	Yes	ZIP	-0.042	1	Yes
Central	Sao Tome and Principe	P	-0.213	Yes	P	0.314	Yes	ZIP	-0.088	Yes				
West	Benin							P	-0.099	Yes	ZIP	-0.061	1	Yes
West	Burkina Faso	P	-0.02	Yes	P	-0.006	Yes	P	-0.056	Yes	ZIP	0.003	2	No
West	Cape Verde	P	-0.016	No	P	0.088	Yes (5%)	P	-0.077	Yes				
West	Chad	P	-0.018	Yes	P	-0.109	Yes	P	-0.033	Yes	ZIP	0.268	2	Yes
West	Côte d'Ivoire	P	-0.03	Yes	P	0.045	Yes	P	-0.075	Yes	ZIP	0.004	2	No
West	Gambia	P	-0.068	Yes	P	0.036	Yes	ZIP	-0.085	Yes				
West	Ghana	P	-0.027	Yes	P	0.034	Yes	P	-0.097	Yes	ZIP	-0.033	1	Yes
West	Guinea	P	-0.013	Yes	P	-0.042	Yes	P	-0.057	Yes	ZIP	-0.069	1	Yes
West	Guinea-Bissau	P	-0.096	Yes	P	0.066	Yes	P	-0.075	Yes	ZIP	0.03	2	No
West	Liberia	P	-0.018	Yes	P	-0.079	Yes	P	-0.065	Yes	ZIP	-0.22	1	Yes
West	Mali	P	-0.002	No	P	-0.036	Yes	P	-0.089	Yes	ZIP	-0.072	1	Yes
West	Mauritania	P	-0.075	Yes	P	-0.035	Yes	P	-0.106	Yes	ZIP	-0.047	1	No
West	Niger	P	0.008	Yes	P	-0.023	Yes	P	-0.048	Yes				
West	Nigeria	P	-0.025	Yes	P	-0.02	Yes	P	-0.05	Yes	ZIP	-0.052	1	Yes
West	Senegal	P	-0.106	Yes	P	0.086	Yes	P	-0.099	Yes	ZIP	-0.026	1	Yes
West	Sierra Leone	P	-0.01	Yes	P	-0.108	Yes	P	-0.056	Yes	ZIP	0.151	2	Yes
West	Togo	P	-0.026	Yes	P	0.013	Yes	P	-0.149	Yes	ZIP	0.039	2	No
South	Botswana	P	-0.188	Yes	P	0.077	Yes	P	-0.099	Yes				
South	Eswatini	P	-0.037	No	P	0.165	Yes	ZIP	-0.122	Yes				
South	Lesotho							P	-0.135	Yes				
South	Namibia	P	-0.095	Yes	P	0.29	Yes	P	-0.136	Yes				
South	South Africa	P	-0.063	Yes (5%)	P	0.019	Yes	P	-0.096	Yes				

193

The Panel Poisson Regression presented in Table 2 showed the effect of Measles and Yellow Fever in each of African Region and Africa as a whole. Malaria incidence increased significantly with measles cases in East Africa while significant negative relationship occurred between the two in West and South African countries ($P<0.001$). Overall, a significant negative relationship existed between malaria incidence and measles cases ($P<0.05$). While a positive relationship existed between malaria incidence and yellow fever cases in West Africa ($P<0.01$), a negative relationship occurred between the two in South Africa ($P<0.001$) (Table 2). Overall, malaria incidence was significantly positively related to yellow fever in Africa ($P<0.05$).

Death due to malaria was significantly negatively related to measles cases in Africa ($P<0.001$). A negative relationship existed between the two West and South African countries ($P<0.001$). Overall, a significant positive relationship existed between death due to malaria and yellow fever in Africa ($P<0.001$) (Table 2).

Table 2. Malaria Incidence and Death due to Random Effect Panel Model

Variables		North	East	Central	West	South	Overall
Malaria incidence	Measles	0.0000074	0.0000053***	0.00000015	-0.00000084***	-0.00057***	-0.00000042**
		(0.52)	(5.73)	(0.65)	(-4.24)	(-6.21)	(-2.86)
	Yellow Fever	0	0.000346	0.0000428	0.000114**	-0.548***	0.0000786**
		(.)	(1.27)	(1.22)	(3.27)	(-4.42)	(3.20)
	trend	-0.0356***	-0.0224***	-0.0308***	-0.0237***	-0.0762***	-0.0262***
		(-6.44)	(-16.11)	(-33.47)	(-34.84)	(-8.11)	(-52.68)
	Constant	4.691***	5.472***	5.960***	5.956***	3.107***	5.653***
		(4.15)	(22.07)	(45.80)	(25.24)	(4.74)	(30.10)
	Observations	27	123	152	264	43	609
Death due to malaria	Measles	-0.000016	-0.000014***	-0.0000031***	-0.0000014***	-0.00038***	-0.0000043***
		(-0.92)	(-59.09)	(-77.37)	(-16.74)	(-20.32)	(-125.04)
	Yellow fever	0	0.00137***	0.0000890***	-0.0000127	-0.356***	0.000156***
		(.)	(15.31)	(9.79)	(-1.10)	(-12.81)	(20.57)
	Trend	-0.0790***	-0.129***	0.00648***	0.00381***	-0.172***	-0.0472***
		(-3.86)	(-377.34)	(20.67)	(11.20)	(-59.53)	(-270.38)
	Constant	5.748***	9.715***	8.456***	7.560***	6.553***	8.567***
		(4.48)	(22.44)	(16.29)	(24.47)	(10.05)	(33.46)
	Observations	15	88	118	224	39	484

t statistics in parentheses

* $p < 0.05$, ** $p < 0.01$, *** $p < 0.001$

Discussion

Malaria is still being actively transmitted across African countries, however, observations from our study showed that the various interventional programmes could be yielding expected results as there was a significant decrease in malaria incidence in about 81% of the observed African countries. Increase insecticide-treated nets (ITNs) coverage across African countries over the years may be responsible for this malaria decline [19,20]. The use of ITNs may result in a decline in the population of indoor *Anopheles* mosquitoes, thus, triggering behavioural changes

in biting and resting of mosquitoes including a shift from an indoor to an outdoor environment [21]. Besides the increase in ITNs coverage, changes in socio-ecological conditions in many malaria-endemic areas of Africa including changes in temperature, deforestation [22], increase use of agricultural pesticides with mosquitocidal properties [23], improved house constructions [24] could result in a decreased density of mosquitoes.

The significant decrease in malaria transmission could have culminated in the 43.5% significant reduction in death associated with malaria in Africa. However, the 52.2% significant increase in malaria-associated deaths in Africa could have resulted from poor access to and low-quality health services, and increased resistance of *Plasmodium falciparum* to first-line drugs [25,26]. While the incidence of malaria may correlate with death associated with the disease, the malaria-associated deaths could be difficult to assess in the actual sense [27,28].

The higher significant decrease in the incidence of measles in many African countries compared with that of yellow fever could portray a probable relaxed public health alert system towards prevention of yellow fever. Importantly, the immunization coverage of yellow fever appeared to be lower than that of measles in many African countries. For example in Nigeria, the mean official yellow fever vaccine (YFV) coverage was 56.3% while that of the first dose of measles-containing vaccine (MCV1) was 65.0%, for the Democratic Republic of Congo, YFV and MCV1 were 80.67 and 87.67% respectively [29,30]. In Togo however, the mean immunization coverage for YFV (82.83%) and MCV1 (82.75%) was similar [31].

While our models showed a generalized positive relationship between the incidence of malaria and measles cases, the factors responsible for this relationship cannot be readily explained at the moment. Nevertheless, a closer look at the differences in the direction of the relationship at the regional levels suggested that malaria transmission could impact measles transmission. For

example, the occurrence of many countries in Eastern Africa with a consistent increase in the incidence of malaria against their Western and Southern counterpart could partly explain the impact of malaria on measles transmission. A recent study from Ghana suggested that malaria can exacerbate inflammatory cytokine responses in chronic hepatitis B patients, thus, increasing susceptibility to liver injury [32]. Another study from the Gambia among HIV-infected pregnant women showed that placental histopathologic malaria was associated with higher concentrations of both peripheral and placental HIV-1 RNA [33]. Studies on the direct relationship of malaria with measles are scarce. One study in Ghanaian pregnant women population, however, associated maternal malaria preventive treatment with increasing infant immunity against measles [34].

The overall positive relationship between malaria and yellow fever could be as a result of co-existence of mosquito vectors of the two diseases in similar ecological zones. In addition to *Aedes* mosquitoes, *Anopheles* mosquitoes have been identified in similar habitats in Africa [35,36]. Although our study showed decreased in the incidence of malaria over the years in many countries in West Africa, the African region is one of the most affected regions of the world contributing more than 30% of the world's malaria-associated deaths [37]. This could be difficult to explain, but complex immune modulation which could influence the susceptibility and immunopathology of yellow fever in malaria-endemic areas may be responsible for the positive relationship in the incidence of the two diseases in the region. The reverse relationship trend between the two diseases in countries in the Southern Africa region may be due to lower malaria transmission in many countries in the region. Overall similar relationship trends observed between malaria incidence and yellow fever/measles were also observed between death due to

malaria and the two vaccine-preventable diseases in Africa. These can be adduced to the aforementioned reasons highlighted for malaria incidence and yellow fever/measles.

Conclusions

Our study showed that significant relationships exist between malaria and yellow fever/measles. The direction of the relationships differed, with yellow fever showing positive and measles negative relationships with malaria incidence and malaria-related deaths, respectively. The relationships observed in this study could be important for the management of malaria and the studied vaccine-preventable diseases. Increase vaccination coverage or malaria treatment could shape the direction of these relationships. Case-control studies are recommended to critically understudy the relationships between malaria, measles and yellow fever.

Conflict of interest

None declared.

Funding

The study received no funding from any organization.

Data availability

The variables data used in this study are available in the WHO database (www.who.int/data/gho).

Authors contributions

Conceptualization – OTO

Data curation – OAO, OTO

Formal analysis – OAO

Investigation – OAO, OTO

Methodology – OAO, OTO

Writing – original draft preparation – OAO, OTO

References

1. World Health Organization. World malaria report 2018. http://apps.who.int/iris/bitstream/handle/10665/275867/9789241565653-eng.pdf?ua-1; Accessed 30 January 2021
2. Winskill P, Walker PG, Cibulskis RE, Ghani AC. Prioritizing the scale-up of interventions for malaria control and elimination. Malar J. 2019;18:122. https://doi.org/10.1186/s12936-019-2755-5
3. Chambers TJ, Monath TP. Preface to the flaviviruses. Adv Virus Res. 2003; 60:ix-xi.
4. Garske T, Van Kerkhove MD, Yactayo S, Ronveaux O, Lewis RF, Staples JE, et al. Yellow Fever in Africa: estimating the burden of disease and impact of mass vaccination from the outbreak and serological data. PLoS Med. 2014;11:e1001638. https://doi.org/10.1371/journal.pmed.1001638
5. Barrett AD, Higgs S. Yellow fever: a disease that has yet to be conquered. Annu Rev Entomol. 2007;52:209-229. https://doi.org/10.1146/annurev.ento.52.110405.091454.
6. Johansson MA, Vasconcelos PF, Staples JE. The whole iceberg: estimating the incidence of yellow fever virus infection from the number of severe cases. Trans R Soc Trop Med Hyg. 2014;108:482-487. https://doi.org/10.1093/trstmh/tru092

7. Verguet S, Jassat W, Hedberg C, Tollman S, Jamison DT, Hofman KJ. Measles control in Sub-Saharan Africa: South Africa as a case study. Vaccine 2012;30:1594–1600. https://doi.org/10.1016/j.vaccine.2011.12.123
8. Oyeyemi OT, Amugo AN. *Plasmodium falciparum* and hepatitis B virus infection in Nigerian urban population. Braz J Infect Dis. 2015a;19: 666-667. https://doi.org/10.1016/j.bjid.2015.07.010.
9. Oyeyemi OT, Amugo AN. Maternal *Plasmodium falciparum* and hepatitis B virus infections in Nigeria. J Dis Global Health 2015b;4(2):84-88.
10. Oyeyemi OT, Etim EJ. Malaria and HIV infections among febrile patients in Lagos, Southwestern, Nigeria. J Interdisciplinary Med. 2020;5:43-47. https://doi.org/10.2478/jim-2020-0011.
11. Sow A, Loucoubar C, Diallo D, Faye O, Ndiaye Y, Saadibou C, et al. Concurrent malaria and arbovirus infections in Kedougou, Southeastern Senegal. Malar J. 2016;15:47. https://doi.org/10.1186/s12936-016-1100-5.
12. Kolawole OM, Irekeola AA, Seriki AA, Bello KE. Investigation of risk factors associated with malaria and yellow fever co-infection among febrile patients in Ilorin, Nigeria. J Med Soc 2018;32:33-39. doi: 10.4103/0972-4958.213955
13. Barry M, Patterson JE, Tirrell S, Cullen MR, Shope RE. The effect of chloroquine prophylaxis on yellow fever vaccine antibody response: comparison of plaque reduction neutralization test and enzyme-linked immunosorbent assay. Am J Trop Med Hyg. 1991; 44:79-82. doi: 10.4269/ajtmh.1991.44.79.
14. Sellers KF, Shmueli G. A flexible regression model for count data. Ann Appl Stat. 2010; 4:943–961.

15. Huang A. Mean-parameterized Conway–Maxwell–Poisson regression models for dispersed counts. Stat Model 2017;17:359–380. https://doi.org/10.1177%2F1471082X17697749
16. Lambert D. Zero-inflated Poisson regression, with an application to defects in manufacturing. Technimetrics 1992;34:1–14.
17. Kibria BMG, Månsson K, Shukur G. Some ridge regression estimators for the zero-inflated Poisson model. J Appl Stat. 2013;40:4:721-735. https://doi.org/10.1080/02664763.2012.752448
18. Nelder JA, Mead R. A simplex method for function minimization. Comp J. 1965;7:308–313. https://doi.org/10.1093/comjnl/7.4.308
19. Okunlola OA, Oyeyemi OT, Lukman AF. Modeling the relationship between malaria prevalence and insecticide-treated bed nets coverage using Leroux prior Bayesian spatial generalized linear mixed models. Epidemiol Health 2021; e2021041. https://doi.org/10.4178/epih.e2021041
20. Bhatt S, Weiss DJ, Cameron E, Bisanzio D, Mappin B, Dalrymple U, et al. The effect of malaria control on Plasmodium falciparum in Africa between 2000 and 2015. Nature 2015;526(7572):207-211. https://doi.org:/10.1038/nature15535.
21. Bogh C, Pedersen EM, Mukoko DA, Ouma JH. Permethrin-impregnated bednet effects on resting and feeding behaviour of lymphatic filariasis vector mosquitoes in Kenya. Med Vet Entomol. 1998;12:52-59. https://doi.org/10.1046/j.1365-2915.1998.00091.x
22. Stresman GH. Beyond temperature and precipitation: Ecological risk factors that modify malaria transmission. Acta Trop 2010;116:167-172. doi:10.1016/j.actatropica.2010.08.

23. Yadouleton AW, Asidi A, Djouaka RF, Braima J, Agossou CD, Akogbeto MC. Development of vegetable farming: a cause of the emergence of insecticide resistance in populations of *Anopheles gambiae* in urban areas of Benin. Malar J. 2009;8:103. https://doi.org/10.1186/1475-2875-8-103.

24. Konradsen F, Amerasinghe P, van der Hoek W, Amerasinghe F, Perera D, Piyaratne M. Strong association between house characteristics and malaria vectors in Sri Lanka. Am J Trop Med Hyg. 2003;68:177-181. https://doi.org/10.4269/ajtmh.2003.68.177.

25. Snow RW, Trape JF, Marsh K. The past, present and future of childhood malaria mortality in Africa. Trends Parasitol 2001;17:593-597. https://doi.org/10.1016/s1471-4922(01)02031-1.

26. Muller O, Traore C, Becher H, Kouyate B. Malaria morbidity, treatment seeking behaviour, and mortality in a cohort of young children in rural Burkina Faso. Trop Med lnt Health 2003;8:290-296. https://doi.org/10.1046/j.1365-3156.2003.01030.x

27. Gemperli A, Vounatsou P, Kleinschmidt I, Bagayoko M, Lengeler C, Smith T. Spatial patterns of infant mortality in Mali: the effect of malaria endemicity. Am J Epidemiol. 2004;159:64-72. https://doi.org/10.1093/aje/kwh001.

28. Omumbo J, Hay S, Guerra C, Snow R. The relationship between the *Plasmodium falciparum* parasite ratio in childhood and climate estimates of malaria transmission in Kenya. Malar J. 2004;3:17. https://doi.org/10.1186/1475-2875-3-17.

29. WHO and UNICEF. Estimates of national immunization coverage – Nigeria 2020a. https://www.who.int/immunization/monitoring_surveillance/data/nga.pdf. Accessed 04 February 2021.

30. WHO and UNICEF (2020c). Estimates of national immunization coverage - Democratic Republic of the Congo. https://www.who.int/immunization/monitoring_surveillance/data/cod.pdf. Accessed 04 February 2021.

31. WHO and UNICEF (2020c). Estimates of national immunization coverage - Togo. https://www.who.int/immunization/monitoring_surveillance/data/tgo.pdf. Accessed 04 February 2021.

32. Anabire NG, Aryee PA, Abdul-Karim A, Quaye O, Awandare GA, Helegbe GK. Impact of malaria and hepatitis B co-infection on clinical and cytokine profiles among pregnant women. PLoS One 2019;14:e0215550. https://doi.org/10.1371/journal.pone.0215550

33. Donovan D, Ariyoshi K, Milligan P, Ota M, Yamuah L, Sarge-Njie R. Maternal plasma viral RNA levels determine marked differences in mother-to-child transmission rates of HIV- 1 and HIV-2 in the Gambia. AIDS 2000;14:441–448. https://doi.org/10.1097/00002030-200003100-00019

34. Owens S, Harper G, Amuasi J, Offei-Larbi G, Ordi J, Brabin BJ. Placental malaria and immunity to infant measles. Arch Dis Child 2006;91:507–508. https://dx.doi.org/10.1136%2Fadc.2005.085274.

35. Addy PA, Esena RK, Atuahene SK. Possible contributing factors to the paucity of yellow fever epidemics in the Ashanti region of Ghana, West Africa. East Afr Med J. 1996;73:3-9.

36. Ondiba IM, Oyieke FA, Athinya DK, Nyamongo IK, Estambale BBA. Larval species diversity, seasonal occurrence and larval habitat preference of mosquitoes transmitting

Rift Valley fever and malaria in Baringo County, Kenya. Parasite Vector 2019;12:295. https://doi.org/10.1186/s13071-019-3557-x.

37. WHO. Malaria Fact Sheet 2020. https://www.who.int/news-room/fact-sheets/detail/malaria. Accessed 04 February 2021.

Figure 1
Click here to access/download;Figure;Fig 1A & B TIF.tif

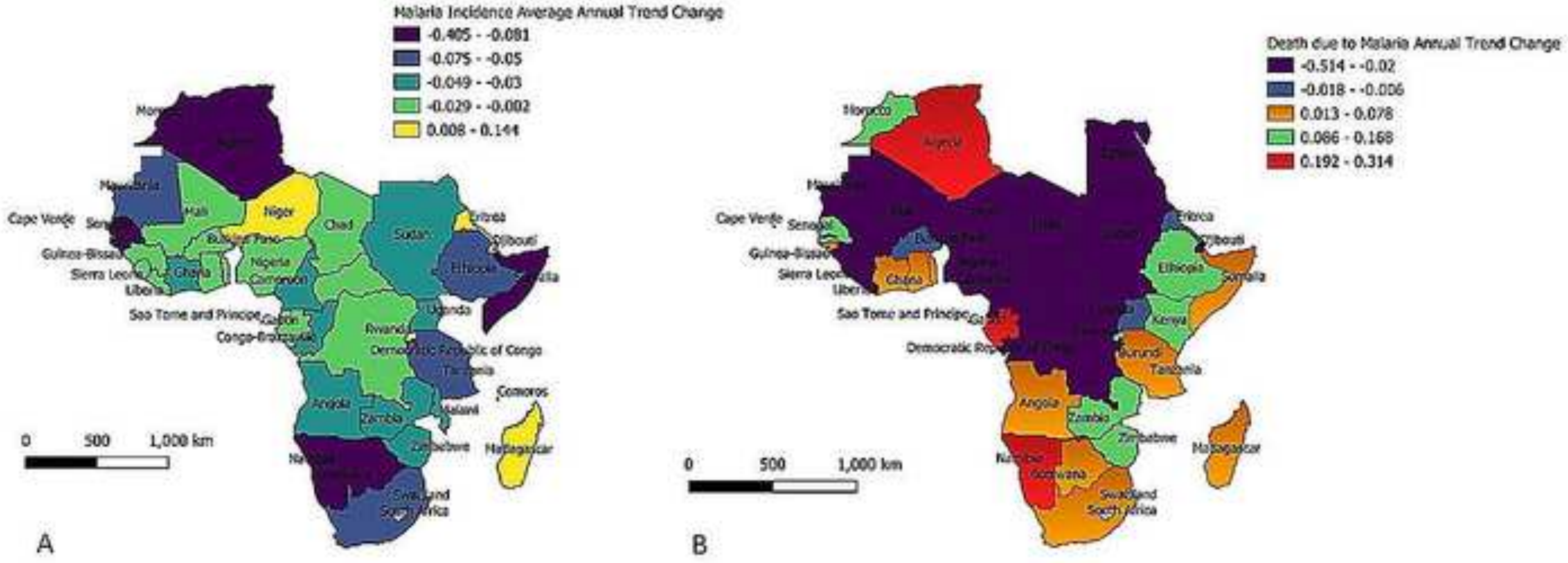

Figure 1: Spatial distribution of malaria (A) and death due to malaria (B) in Africa

Figure 2 Click here to access/download;Figure;Fig. 2A & B TIF.tif

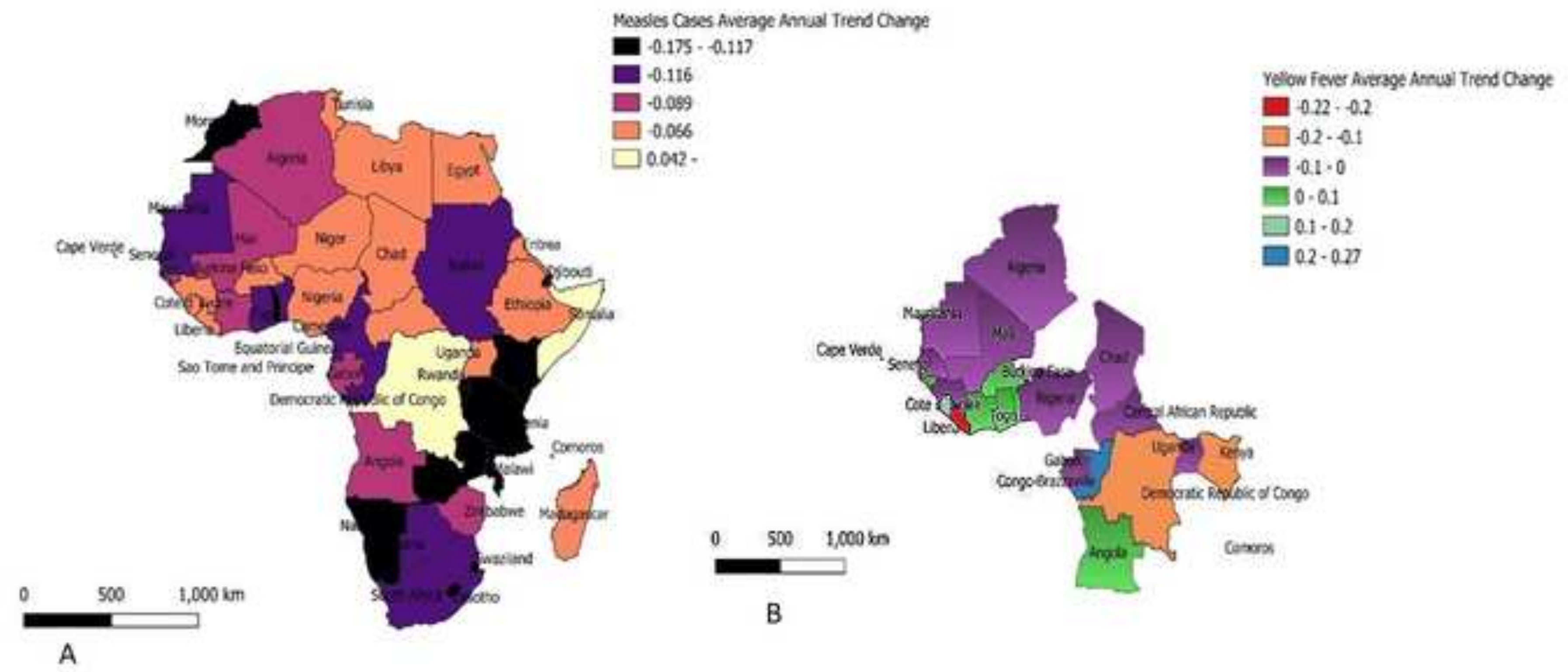

Figure 2: Spatial distribution of measles (A) and yellow fever cases (B) in Africa

Click here to access/download
Supporting Information
Supplementary Data.xlsx

Malaria transmission in Africa: its relationship with yellow fever and measles

Running head - Malaria and vaccine preventable diseases

Oluyemi A. Okunlola[1] and Oyetunde T. Oyeyemi[2*]

[1]Department of Mathematics, University of Medical Sciences, Ondo, Ondo State, Nigeria

[2]Department of Biological Sciences, University of Medical Sciences, Ondo, Ondo State, Nigeria

[*] Corresponding author: Oyetunde T. Oyeyemi

Address: Department of Biological Sciences, University of Medical Sciences, Ondo, Ondo State, Nigeria.

E-mail address: ooyeyemi@unimed.edu.ng

Phone number: +2348163546787

Conflict of interest statement for all authors: authors declared no conflict of interest

Access to data: All authors had access to the data and a role in writing the manuscript

Article type: Research Article

Key words; Malaria, Vaccine-preventable diseases, Modelling, Relationships, Africa

// Abstract

Background

Malaria has been strongly linked to the transmission and pathophysiology of some viral diseases. Few studies are however available on the possible relationships between malaria and vaccine-preventable diseases. The study aims to evaluate the relationships between the incidence of malaria and cases of measles and yellow fever in Africa.

Methods

The malaria incidence, death due to malaria, measles and yellow fever data were sourced from the WHO database. Poisson and zero-inflated time-trend regression were used to model the relationships between malaria and the two vaccine-preventable diseases.

Results

A significant negative relationship existed between malaria incidence and measles cases (P<0.05), however, malaria showed a positive relationship with yellow fever (P<0.05). The relationships between death due to malaria and measles/yellow fever cases followed similar trends but with a higher level of statistical significance (P<0.001).

Conclusions

The relationships observed in this study could be important for the management of malaria and the studied vaccine-preventable diseases. Increase vaccination coverage and/or malaria treatment could modulate the direction of these relationships.

Key words: Malaria, Vaccine-preventable diseases, Modelling, Relationships, Africa

Introduction

Malaria is the most important parasitic disease in the tropical and sub-tropical regions of the world. In 2017, an estimated 219 million malaria cases and 435 000 malarial-associated deaths were recorded globally [1]. Africa is the most affected region of the world; accounting for more than 90% malaria cases and associated deaths [1]. The recommended malaria interventions include prompt detection of infection, treatment of clinical and severe malaria, vector control, and chemoprevention in high-risk groups [2].

Yellow fever is a vector-borne disease caused by the yellow fever virus of the genus *Flavivirus* [3]. With estimated 51 000 to 380 000 severe cases of yellow fever, and 19 000 to 180 000 deaths per year in Africa [4], the continent is the epicenter of the disease recording 90% of the total global cases of the disease [5]. Approximately half of the infected populations are asymptomatic and a further third suffer from a mild illness. Only about 12% of yellow fever infections present a severe disease in the form of jaundice and/or haemorrhage with 30-60% death rate [6]. Measles, like malaria and yellow fever, is another disease with serious public health implications for children. Although the global transmission of measles has been greatly reduced by vaccination about 60 years ago, it continues to be one of the major causes of mortality among children in sub-Saharan Africa [7].

Previous studies have related malaria with some viral infections, especially HIV and hepatitis [8-10], and these studies suggested the possible impact of malaria on the dynamics of transmission and morbidities associated with viral infections. Despite the dreadfulness of malaria and viral agents in the current study, studies on concomitant presentation of these diseases are very rare. Although few epidemiological studies are available for malaria and yellow fever [11,12], none is available for malaria and measles. Other studies involving the triads included the effect of

chloroquine prophylaxis on yellow fever vaccine antibody response [13], and impact of placental malaria on infant immunity to measles.

The aim of the current study is to determine the magnitude and direction of the trend of malaria incidence, death due to malaria, measles cases and yellow fever cases in selected African countries. Also to investigate the effect of measles and yellow fever cases on malaria incidence and death due to malaria, respectively. ~~Because all the studied variables are cases and rates which are regarded as count data, a Panel Poisson regression model was adopted to understudy these relationships. Where there are observed excess zero in the cases of event considered in the study, a zero-inflated Poisson (ZIP) regression that is robust to the occurrence of zero is adopted to achieve our specified objectives~~. This study could have implications on the control of malaria and these vaccine-preventable diseases.

Material and methods

Data and scope

The variables data examined in the study were sourced from the WHO database (www.who.int/data/gho) from 2000-2018 for both malaria incidence and death due to malaria while the year 1980-2019 was used for measles and yellow fever cases for all African countries with a well-filled data on the mentioned variables. Consequently, 39 countries were included for malaria incidence, 28 countries for death due to malaria, 52 countries for measles and 30 countries for yellow fever annual trend change model.

Regression analyses

Count data are known to be discrete variables and not continuous, thus, the traditional regression model that assumes that outcome or response variable comes from a normal distribution is not

tenable bearing in mind that discrete variable is highly skewed. This study used a discrete probability distribution that treats the response variable of count observation as a Poisson process with probability mass function given as $f(y) = \frac{e^{-\mu}\mu^{y}}{y!}, 0 \leq y \leq \infty$ (1)

This bound indicates that the distribution takes on nonnegative random numbers. The quantity λ is the mean of the Poisson distribution. This distribution has a unique property of equi-dispersion that the mean, $\lambda = E(y)$ is equal to variance, $V(y)$. However, the assumption of equi-dispersion is highly restrictive and hardly satisfied in real-life application as shown here that the mean each variable considered is not equal to the variance. Two major problems that are associated with Poisson regression are over-dispersion and under-dispersion which connote situations where $V(y) > E(y)$ and $V(y) < E(y)$, respectively. Sellers and Shmueli [14] provided a more general count distribution that captured a wide range of dispersion which they titled Conway–Maxwell-Poisson (COM-Poisson) and Huang [15] provided a COM-Poisson Double Generalized Linear Model that addressed the problem of both under and overdispersion simultaneous.

Poisson regression is achieved by expressing λ in equation (1) as a function of some explanatory variables through a log link function. This can be mathematically expressed as:

$$\lambda_i = X'\beta \text{ or } \quad \mu_i = \exp(X'\beta) \tag{2}$$

Given the density function of equation (1) type and set of independent variable X_i, the maximum likelihood estimates of regression parameter β is defined as;

$$log_e\, L(\beta) = \sum\{Y_i X'\beta - \exp(X'\beta) - \ln(Y!)\} \tag{3}$$

Count data most often have excess zero cases and if not adjusted for can lead to inaccurate regression estimate and misleading conclusion. Lambert [16] developed a zero-inflated Poisson (ZIP) regression that corrects for the occurrence of excess zero cases in count data. The ZIP has

become a popular choice in empirical problems relating to count data. The ZIP model is often appealing, first, since it divides the dependent variable into two subpopulations where the first one takes on the value of zero with probability π_i while the second one is Poisson distributed with probability $1 - \pi_i$ [17]. Due to this setup, the ZIP model may be used when the data contain an excess amount of zeros. Second, this model is popular because it accounts for overdispersion (meaning that the variance of the dependent variable exceeds the mean value), which leads to an underestimation of the variance of the estimated coefficients when applying the standard Poisson regression model [17]. The ZIP divides the count data into two non-overlapping subpopulations using the equation:

$$y_i = \begin{cases} 0 & with\ porbability\ \pi_i \\ P_0(\mu_i) & with\ porbability\ 1 - \pi_i \end{cases} \text{ and } \pi_i = \frac{\exp(q_i'\gamma)}{1+\exp(q_i'\gamma)} \quad (3)$$

In this expression q_i is the ith row of the Q, which is the data matrix for the logit model and γ corresponds to a $(p + 1) \times 1$ vector of coefficients. Also, $\mu_i = \exp(x_i'\beta)$, where x_i is the ith row of X, which is the data matrix for the ZIP model while β corresponds to a $(p + 1) \times 1$ vector of coefficients. The most common method of estimating β is to apply the ML method. By defining 1*(yi* = 0*)* as an indicator function that takes on the value of 1 if $y_i = 0$, the following joint likelihood should then be maximized:

$$L(\beta, \gamma) = \sum_{i=1}^{N}(l(y_i = 0)(\log(\exp(q_i'\gamma)) + \exp(-\exp(-\exp(X_i'\beta)))) + \sum_{i=1}^{N}(1 - l(y_i = 0)(y_i', x_i'\beta - \exp(x_i'\beta - \exp(x_i'\beta))] - \sum_{i=1}^{N}\log(1 + \exp(q_i'\gamma)) \quad (4)$$

This complex likelihood function is estimated through the modification of the simplex method of Nelder and Mead [18] proposed by Kibria et al. [17] in which the start-up values come from the iterative weighted least-squares algorithm of the individual Poisson and logit estimation. The modification to this method applied the estimator specified in (5).

$$\hat{\beta}_{RR} = (X`\widehat{W}X + kI)^{-1}X`\widehat{W}X\hat{\beta}_{ML} \quad (5)$$

$\hat{\beta}_{ML}$ is the estimate obtained using the simplex method. Furthermore, the shrinkage parameter k may take on values between zero and infinity, and when k equals zero, we have $\hat{\beta}_{RR} = \hat{\beta}_{ML}$. When k is greater than zero, we have $||\hat{\beta}_{RR}|| < ||\hat{\beta}_{ML}||$. Since $\hat{\beta}_{ML}$ is, on average, too long in the presence of multicollinearity, $\hat{\beta}_{RR}$ is expected to perform better than $\hat{\beta}_{ML}$.

Statistical analysis

The statistical analysis was carried out using five statistical software packages. SPSS version 23 was used for data management, cleaning and descriptive analysis. STATA version 13 and R version 4.0.3 were used to estimate the Poisson and zero-inflated Poisson (ZIP) regression model. GeoDA 1.9 was used to prepare the shapefile and merging of the estimated annual average change (EAAC) while QGIS version 3.14 was used for geospatial mapping of the EAAC of each variable.

Results

Time trend Poisson and zero-inflated Poisson model was fitted for malaria incidences (47 countries), death due to malaria (46 countries), measles cases (54 countries) and yellow fever cases (22 countries). Poisson regression was used for 46(97.87%), 46(100.0%) and 47(87.04%) for malaria incidence, death due to malaria and measles cases while zero-inflated Poisson was used in 1(2.13%), 7(12.06%) and 22(100.0%) countries where there were clear occurrences of excess zero in the malaria incidence, measles cases and yellow fever cases respectively. Malaria incidence reduced significantly in 38(80.9%) countries, increased significantly in 5(10.6%) and increased insignificantly in 4(8.51%) of out the 47 countries observed in the study

over time. Death due to malaria decreased significantly in 20(43.5%) countries but increased significantly in 24(52.2%) of the 46 countries included for the study over time. Also, of the total 54 countries studied for measles cases, 49(90.7%) showed significant decrease trend, 4(7.4%) significant increase trend and 1(1.85%) insignificant time trend coefficient. Yellow fever cases decreased significantly in 13(59.1%), increased significantly in 3(13.6%) countries while the remaining countries 6(27.27%) showed an insignificant trend (Table 1).

The spatial distribution of malaria incidence in African countries is presented in Fig 1. Malaria incidence average annual trend decreased significantly in several African countries except in Algeria, Cape Verde, Mali and Eswatini with time trend regressions -0.088, -0.016, -0.002 and -0.037, respectively, which showed a non-significant trend (P>0.05). A significant increase in malaria incidence was recorded in Djibouti (0.144), Eritrea (0.046), Madagascar (0.024), Rwanda (0.078), and Niger (0.008) (P<0.05) (Table 1).

Fig 1. Spatial Distribution of Malaria (A) and Death due to Malaria (B) in Africa.

The spatial distribution of malaria-associated death in African countries is presented in Fig 1. Algeria with trend regression (0.257) recorded the highest increase in death due to malaria while Egypt (-0.514), on the other hand, recorded the highest decrease in death due to malaria (P<0.05). Several countries in East Africa recorded a significant increase in malaria-associated death while many countries in Central Africa however, showed a significant decrease in malaria-associated death (P<0.05). All the countries in the South African region recorded a significant increase in death due to malaria (P<0.05) (Table 1).

The spatial distribution of measles in African countries is presented in Fig 2. Majority of the African countries recorded a significant decrease in measles cases except in Mauritius (0.042),

Somalia (0.063), South Sudan (0.058), and Democratic Republic of Congo (0.062) (P<0.05). Seychelles (-0.006) showed a non-significant decrease in measles cases annual trend (P>0.05) (Table 1). The spatial distribution of yellow fever cases is presented in Fig 2. Chad with trend regression 0.268 recorded the highest significant increase in yellow fever cases while Congo (-0.236) showed the highest significant decrease in yellow fever cases in Africa (P<0.05). The Central Africa Republic (-0.017), Burkina Faso (0.003), Côte d'Ivoire (0.004), Guinea-Bissau (0.03), Mauritania (-0.047), and Togo (0.039) recorded non-significant yellow fever cases trends (Table 1).

Fig 2. Spatial Distribution of Measles (A) and Yellow Fever Cases (B) in Africa.

Table 1. Poisson and Zero Inflated Time Trend Regression

		Malaria Incidence			Death due to Malaria			Measles Cases			Yellow fever Cases			
Region	Country	Method	Estimate	Significance	Method	Estimate	~~Significance~~	Method	Estimate	Significance	Method	Estimate	YFTD	Significance
North	Algeria	P	-0.088	No	P	0.257	Yes (5%)	P	-0.082	Yes				
North	Egypt				P	-0.514	Yes (5%)	P	-0.045	Yes				
North	Libya							P	-0.041	Yes				
North	Morocco	ZIP	-0.405	Yes	P	0.13	No	P	-0.17	Yes				
North	Sudan	P	-0.046	Yes	P	-0.144	Yes	P	-0.111	Yes				
North	Tunisia							P	-0.064	Yes				
East	Burundi	P	-0.05	Yes	P	-0.171	Yes	P	-0.135	Yes				
East	Comoros	P	-0.084	Yes				ZIP	-0.087	Yes				
East	Djibouti	P	0.144	Yes	P	-0.055	No	P	-0.117	Yes				
East	Eritrea	P	0.046	Yes	P	-0.018	Yes	P	-0.061	Yes				
East	Ethiopia	P	-0.074	Yes	P	0.093	Yes	P	-0.039	Yes				
East	Kenya	P	-0.08	Yes	P	0.165	Yes	P	-0.175	Yes	ZIP	-0.119	1	Yes
East	Madagascar	P	0.024	Yes	P	0.019	Yes	P	-0.042	Yes				
East	Malawi	P	-0.036	Yes				P	-0.126	Yes				
East	Mauritius							ZIP	0.042	Yes				
East	Mozambique	P	-0.025	Yes	P	0.125	Yes	P	-0.066	Yes				
East	Rwanda	P	0.078	Yes	P	0.168	Yes	P	-0.168	Yes				
East	Seychelles							ZIP	-0.006	No				
East	Somalia	P	-0.104	Yes	P	0.078	Yes	P	0.063	Yes				
East	South Sudan	P	-0.029	Yes	P	-0.255	Yes	P	0.058	Yes				
East	Uganda	P	-0.038	Yes	P	-0.011	Yes	P	-0.04	Yes	ZIP	-0.071	1	Yes
East	United Republic of Tanzania	P	-0.07	Yes	P	0.013	Yes	P	-0.139	Yes				
East	Zambia	P	-0.049	Yes	P	0.101	Yes	P	-0.128	Yes				
East	Zimbabwe	P	-0.033	Yes	P	0.145	Yes	ZIP	-0.077	Yes				
Central	Angola	P	-0.035	Yes	P	0.035	Yes	P	-0.084	Yes	ZIP	0.11	2	Yes
Central	Cameroon	P	-0.034	Yes	P	-0.026	Yes	P	-0.107	Yes	ZIP	-0.032	1	Yes
Central	Central African Republic	P	-0.016	Yes	P	-0.165	Yes	P	-0.065	Yes	ZIP	-0.017	1	No

Central	Congo	P	-0.033	Yes	P	-0.078	Yes	P	-0.116	Yes	ZIP	-0.236	1	Yes
Central	Democratic Republic of the Congo	P	-0.028	Yes	P	-0.075	Yes	P	0.062	Yes	ZIP	-0.136	1	Yes
Central	Equatorial Guinea	P	-0.02	Yes	P	-0.161	Yes	P	-0.101	Yes				
Central	Gabon	P	-0.007	Yes (5%)	P	0.192	Yes	P	-0.085	Yes	ZIP	-0.042	1	Yes
Central	Sao Tome and Principe	P	-0.213	Yes	P	0.314	Yes	ZIP	-0.088	Yes				
West	Benin							P	-0.099	Yes	ZIP	-0.061	1	Yes
West	Burkina Faso	P	-0.02	Yes	P	-0.006	Yes	P	-0.056	Yes	ZIP	0.003	2	No
West	Cape Verde	P	-0.016	No	P	0.088	Yes (5%)	P	-0.077	Yes				
West	Chad	P	-0.018	Yes	P	-0.109	Yes	P	-0.033	Yes	ZIP	0.268	2	Yes
West	Côte d'Ivoire	P	-0.03	Yes	P	0.045	Yes	P	-0.075	Yes	ZIP	0.004	2	No
West	Gambia	P	-0.068	Yes	P	0.036	Yes	ZIP	-0.085	Yes				
West	Ghana	P	-0.027	Yes	P	0.034	Yes	P	-0.097	Yes	ZIP	-0.033	1	Yes
West	Guinea	P	-0.013	Yes	P	-0.042	Yes	P	-0.057	Yes	ZIP	-0.069	1	Yes
West	Guinea-Bissau	P	-0.096	Yes	P	0.066	Yes	P	-0.075	Yes	ZIP	0.03	2	No
West	Liberia	P	-0.018	Yes	P	-0.079	Yes	P	-0.065	Yes	ZIP	-0.22	1	Yes
West	Mali	P	-0.002	No	P	-0.036	Yes	P	-0.089	Yes	ZIP	-0.072	1	Yes
West	Mauritania	P	-0.075	Yes	P	-0.035	Yes	P	-0.106	Yes	ZIP	-0.047	1	No
West	Niger	P	0.008	Yes	P	-0.023	Yes	P	-0.048	Yes				
West	Nigeria	P	-0.025	Yes	P	-0.02	Yes	P	-0.05	Yes	ZIP	-0.052	1	Yes
West	Senegal	P	-0.106	Yes	P	0.086	Yes	P	-0.099	Yes	ZIP	-0.026	1	Yes
West	Sierra Leone	P	-0.01	Yes	P	-0.108	Yes	P	-0.056	Yes	ZIP	0.151	2	Yes
West	Togo	P	-0.026	Yes	P	0.013	Yes	P	-0.149	Yes	ZIP	0.039	2	No
South	Botswana	P	-0.188	Yes	P	0.077	Yes	P	-0.099	Yes				
South	Eswatini	P	-0.037	No	P	0.165	Yes	ZIP	-0.122	Yes				
South	Lesotho							P	-0.135	Yes				
South	Namibia	P	-0.095	Yes	P	0.29	Yes	P	-0.136	Yes				
South	South Africa	P	-0.063	Yes (5%)	P	0.019	Yes	P	-0.096	Yes				

The Panel Poisson Regression presented in Table 2 showed the effect of Measles and Yellow Fever in each of African Region and Africa as a whole. Malaria incidence increased significantly with measles cases in East Africa while significant negative relationship occurred between the two in West and South African countries (P<0.001). Overall, a significant negative relationship existed between malaria incidence and measles cases (P<0.05). While a positive relationship existed between malaria incidence and yellow fever cases in West Africa (P<0.01), a negative relationship occurred between the two in South Africa (P<0.001) (Table 2). Overall, malaria incidence was significantly positively related to yellow fever in Africa (P<0.05).

Death due to malaria was significantly negatively related to measles cases in Africa (P<0.001). A negative relationship existed between the two West and South African countries (P<0.001). Overall, a significant positive relationship existed between death due to malaria and yellow fever in Africa (P<0.001) (Table 2).

Table 2. Malaria Incidence and Death due to Random Effect Panel Model

Variables		North	East	Central	West	South	Overall
Malaria incidence	Measles	0.0000074	0.0000053***	0.00000015	-0.00000084***	-0.00057***	-0.00000042**
		(0.52)	(5.73)	(0.65)	(-4.24)	(-6.21)	(-2.86)
	Yellow Fever	0	0.000346	0.0000428	0.000114**	-0.548***	0.0000786**
		(.)	(1.27)	(1.22)	(3.27)	(-4.42)	(3.20)
	trend	-0.0356***	-0.0224***	-0.0308***	-0.0237***	-0.0762***	-0.0262***
		(-6.44)	(-16.11)	(-33.47)	(-34.84)	(-8.11)	(-52.68)
	Constant	4.691***	5.472***	5.960***	5.956***	3.107***	5.653***
		(4.15)	(22.07)	(45.80)	(25.24)	(4.74)	(30.10)
	Observations	27	123	152	264	43	609
Death due to malaria	Measles	-0.000016	-0.000014***	-0.0000031***	-0.0000014***	-0.00038***	-0.0000043***
		(-0.92)	(-59.09)	(-77.37)	(-16.74)	(-20.32)	(-125.04)
	Yellow fever	0	0.00137***	0.0000890***	-0.0000127	-0.356***	0.000156***
		(.)	(15.31)	(9.79)	(-1.10)	(-12.81)	(20.57)
	Trend	-0.0790***	-0.129***	0.00648***	0.00381***	-0.172***	-0.0472***
		(-3.86)	(-377.34)	(20.67)	(11.20)	(-59.53)	(-270.38)
	Constant	5.748***	9.715***	8.456***	7.560***	6.553***	8.567***
		(4.48)	(22.44)	(16.29)	(24.47)	(10.05)	(33.46)
	Observations	15	88	118	224	39	484

t statistics in parentheses
* $p < 0.05$, ** $p < 0.01$, *** $p < 0.001$

Discussion

Malaria is still being actively transmitted across African countries, however, observations from our study showed that the various interventional programmes could be yielding expected results as there was a significant decrease in malaria incidence in about ~~81% of the observed African countries~~. Increase insecticide-treated nets (ITNs) coverage across African countries over the years may be responsible for this malaria decline [19,20]. The use of ITNs may result in a decline in the population of indoor *Anopheles* mosquitoes, thus, ~~triggering behavioural changes~~

in biting and resting of mosquitoes including a shift from an indoor to an outdoor environment [21]. Besides the increase in ITNs coverage, changes in socio-ecological conditions in many malaria-endemic areas of Africa including changes in temperature, deforestation [22], increase use of agricultural pesticides with mosquitocidal properties [23], improved house constructions [24] could result in a decreased density of mosquitoes.

The significant decrease in malaria transmission could have culminated in the 43.5% significant reduction in death associated with malaria in Africa. However, the 52.2% significant increase in malaria-associated deaths in Africa could have resulted from poor access to and low-quality health services, and increased resistance of *Plasmodium falciparum* to first-line drugs [25,26]. While the incidence of malaria may correlate with death associated with the disease, the malaria-associated deaths could be difficult to assess in the actual sense [27,28].

The higher significant decrease in the incidence of measles in many African countries compared with that of yellow fever could portray a probable relaxed public health alert system towards prevention of yellow fever. Importantly, the immunization coverage of yellow fever appeared to be lower than that of measles in many African countries. For example in Nigeria, the mean official yellow fever vaccine (YFV) coverage was 56.3% while that of the first dose of measles-containing vaccine (MCV1) was 65.0%, for the Democratic Republic of Congo, YFV and MCV1 were 80.67 and 87.67% respectively [29,30]. In Togo however, the mean immunization coverage for YFV (82.83%) and MCV1 (82.75%) was similar [31].

While our models showed a ~~generalized positive relationship between the incidence of malaria and measles cases,~~ the factors responsible for this relationship cannot be readily explained at the moment. Nevertheless, a closer look at the differences in the direction of the relationship at the regional levels suggested that malaria transmission could impact measles transmission. For

example, the occurrence of many countries in Eastern Africa with a consistent increase in the incidence of malaria against their Western and Southern counterpart could partly explain the impact of malaria on measles transmission. A recent study from Ghana suggested that malaria can exacerbate inflammatory cytokine responses in chronic hepatitis B patients, thus, increasing susceptibility to liver injury [32]. Another study from the Gambia among HIV-infected pregnant women showed that placental histopathologic malaria was associated with higher concentrations of both peripheral and placental HIV-1 RNA [33]. Studies on the direct relationship of malaria with measles are scarce. One study in Ghanaian pregnant women population, however, associated maternal malaria preventive treatment with increasing infant immunity against measles [34].

The overall positive relationship between malaria and yellow fever could be as a result of co-existence of mosquito vectors of the two diseases in similar ecological zones. In addition to *Aedes* mosquitoes, *Anopheles* mosquitoes have been identified in similar habitats in Africa [35,36]. Although our study showed decreased in the incidence of malaria over the years in many countries in West Africa, the African region is one of the most affected regions of the world contributing more than 30% of the world's malaria-associated deaths [37]. This could be difficult to explain, but complex immune modulation which could influence the susceptibility and immunopathology of yellow fever in malaria-endemic areas may be responsible for the positive relationship in the incidence of the two diseases in the region. The reverse relationship trend between the two diseases in countries in the Southern Africa region may be due to lower malaria transmission in many countries in the region. Overall similar relationship trends observed between malaria incidence and yellow fever/measles were also observed between death due to

malaria and the two vaccine-preventable diseases in Africa. These can be adduced to the aforementioned reasons highlighted for malaria incidence and yellow fever/measles.

Conclusions

Our study showed that significant relationships exist between malaria and yellow fever/measles. The direction of the relationships differed, with yellow fever showing positive and measles negative relationships with malaria incidence and malaria-related deaths, respectively. The relationships observed in this study could be important for the management of malaria and the studied vaccine-preventable diseases. Increase vaccination coverage or malaria treatment could shape the direction of these relationships. Case-control studies are recommended to critically understudy the relationships between malaria, measles and yellow fever.

Conflict of interest

None declared.

Funding

The study received no funding from any organization.

Data availability

The variables data used in this study are available in the WHO database (www.who.int/data/gho).

Authors contributions

Conceptualization – OTO

Data curation – OAO, OTO

Formal analysis – OAO

Investigation – OAO, OTO

Methodology – OAO, OTO

Writing – original draft preparation – OAO, OTO

References

1. World Health Organization. World malaria report 2018. http://apps.who.int/iris/bitstream/handle/10665/275867/9789241565653-eng.pdf?ua-1; Accessed 30 January 2021
2. Winskill P, Walker PG, Cibulskis RE, Ghani AC. Prioritizing the scale-up of interventions for malaria control and elimination. Malar J. 2019;18:122. https://doi.org/10.1186/s12936-019-2755-5
3. Chambers TJ, Monath TP. Preface to the flaviviruses. Adv Virus Res. 2003; 60:ix-xi.
4. Garske T, Van Kerkhove MD, Yactayo S, Ronveaux O, Lewis RF, Staples JE, et al. Yellow Fever in Africa: estimating the burden of disease and impact of mass vaccination from the outbreak and serological data. PLoS Med. 2014;11:e1001638. https://doi.org/10.1371/journal.pmed.1001638
5. Barrett AD, Higgs S. Yellow fever: a disease that has yet to be conquered. Annu Rev Entomol. 2007;52:209-229. https://doi.org/10.1146/annurev.ento.52.110405.091454.
6. Johansson MA, Vasconcelos PF, Staples JE. The whole iceberg: estimating the incidence of yellow fever virus infection from the number of severe cases. Trans R Soc Trop Med Hyg. 2014;108:482-487. https://doi.org/10.1093/trstmh/tru092

7. Verguet S, Jassat W, Hedberg C, Tollman S, Jamison DT, Hofman KJ. Measles control in Sub-Saharan Africa: South Africa as a case study. Vaccine 2012;30:1594–1600. https://doi.org/10.1016/j.vaccine.2011.12.123
8. Oyeyemi OT, Amugo AN. *Plasmodium falciparum* and hepatitis B virus infection in Nigerian urban population. Braz J Infect Dis. 2015a;19: 666-667. https://doi.org/10.1016/j.bjid.2015.07.010.
9. Oyeyemi OT, Amugo AN. Maternal *Plasmodium falciparum* and hepatitis B virus infections in Nigeria. J Dis Global Health 2015b;4(2):84-88.
10. Oyeyemi OT, Etim EJ. Malaria and HIV infections among febrile patients in Lagos, Southwestern, Nigeria. J Interdisciplinary Med. 2020;5:43-47. https://doi.org/10.2478/jim-2020-0011.
11. Sow A, Loucoubar C, Diallo D, Faye O, Ndiaye Y, Saadibou C, et al. Concurrent malaria and arbovirus infections in Kedougou, Southeastern Senegal. Malar J. 2016;15:47. https://doi.org/10.1186/s12936-016-1100-5.
12. Kolawole OM, Irekeola AA, Seriki AA, Bello KE. Investigation of risk factors associated with malaria and yellow fever co-infection among febrile patients in Ilorin, Nigeria. J Med Soc 2018;32:33-39. doi: 10.4103/0972-4958.213955
13. Barry M, Patterson JE, Tirrell S, Cullen MR, Shope RE. The effect of chloroquine prophylaxis on yellow fever vaccine antibody response: comparison of plaque reduction neutralization test and enzyme-linked immunosorbent assay. Am J Trop Med Hyg. 1991; 44:79-82. doi: 10.4269/ajtmh.1991.44.79.
14. Sellers KF, Shmueli G. A flexible regression model for count data. Ann Appl Stat. 2010; 4:943–961.

15. Huang A. Mean-parameterized Conway–Maxwell–Poisson regression models for dispersed counts. Stat Model 2017;17:359–380. https://doi.org/10.1177%2F1471082X17697749

16. Lambert D. Zero-inflated Poisson regression, with an application to defects in manufacturing. Technimetrics 1992;34:1–14.

17. Kibria BMG, Månsson K, Shukur G. Some ridge regression estimators for the zero-inflated Poisson model. J Appl Stat. 2013;40:4:721-735. https://doi.org/10.1080/02664763.2012.752448

18. Nelder JA, Mead R. A simplex method for function minimization. Comp J. 1965;7:308–313. https://doi.org/10.1093/comjnl/7.4.308

19. Okunlola OA, Oyeyemi OT, Lukman AF. Modeling the relationship between malaria prevalence and insecticide-treated bed nets coverage using Leroux prior Bayesian spatial generalized linear mixed models. Epidemiol Health 2021; e2021041. https://doi.org/10.4178/epih.e2021041

20. Bhatt S, Weiss DJ, Cameron E, Bisanzio D, Mappin B, Dalrymple U, et al. The effect of malaria control on Plasmodium falciparum in Africa between 2000 and 2015. Nature 2015;526(7572):207-211. https://doi.org:/10.1038/nature15535.

21. Bogh C, Pedersen EM, Mukoko DA, Ouma JH. Permethrin-impregnated bednet effects on resting and feeding behaviour of lymphatic filariasis vector mosquitoes in Kenya. Med Vet Entomol. 1998;12:52-59. https://doi.org/10.1046/j.1365-2915.1998.00091.x

22. Stresman GH. Beyond temperature and precipitation: Ecological risk factors that modify malaria transmission. Acta Trop 2010;116:167-172. doi:10.1016/j.actatropica.2010.08.

23. Yadouleton AW, Asidi A, Djouaka RF, Braima J, Agossou CD, Akogbeto MC. Development of vegetable farming: a cause of the emergence of insecticide resistance in populations of *Anopheles gambiae* in urban areas of Benin. Malar J. 2009;8:103. https://doi.org/10.1186/1475-2875-8-103.

24. Konradsen F, Amerasinghe P, van der Hoek W, Amerasinghe F, Perera D, Piyaratne M. Strong association between house characteristics and malaria vectors in Sri Lanka. Am J Trop Med Hyg. 2003;68:177-181. https://doi.org/10.4269/ajtmh.2003.68.177.

25. Snow RW, Trape JF, Marsh K. The past, present and future of childhood malaria mortality in Africa. Trends Parasitol 2001;17:593-597. https://doi.org/10.1016/s1471-4922(01)02031-1.

26. Muller O, Traore C, Becher H, Kouyate B. Malaria morbidity, treatment seeking behaviour, and mortality in a cohort of young children in rural Burkina Faso. Trop Med lnt Health 2003;8:290-296. https://doi.org/10.1046/j.1365-3156.2003.01030.x

27. Gemperli A, Vounatsou P, Kleinschmidt I, Bagayoko M, Lengeler C, Smith T. Spatial patterns of infant mortality in Mali: the effect of malaria endemicity. Am J Epidemiol. 2004;159:64-72. https://doi.org/10.1093/aje/kwh001.

28. Omumbo J, Hay S, Guerra C, Snow R. The relationship between the *Plasmodium falciparum* parasite ratio in childhood and climate estimates of malaria transmission in Kenya. Malar J. 2004;3:17. https://doi.org/10.1186/1475-2875-3-17.

29. WHO and UNICEF. Estimates of national immunization coverage – Nigeria 2020a. https://www.who.int/immunization/monitoring_surveillance/data/nga.pdf. Accessed 04 February 2021.

30. WHO and UNICEF (2020c). Estimates of national immunization coverage - Democratic Republic of the Congo. https://www.who.int/immunization/monitoring_surveillance/data/cod.pdf. Accessed 04 February 2021.

31. WHO and UNICEF (2020c). Estimates of national immunization coverage - Togo. https://www.who.int/immunization/monitoring_surveillance/data/tgo.pdf. Accessed 04 February 2021.

32. Anabire NG, Aryee PA, Abdul-Karim A, Quaye O, Awandare GA, Helegbe GK. Impact of malaria and hepatitis B co-infection on clinical and cytokine profiles among pregnant women. PLoS One 2019;14:e0215550. https://doi.org/10.1371/journal.pone.0215550

33. Donovan D, Ariyoshi K, Milligan P, Ota M, Yamuah L, Sarge-Njie R. Maternal plasma viral RNA levels determine marked differences in mother-to-child transmission rates of HIV- 1 and HIV-2 in the Gambia. AIDS 2000;14:441–448. https://doi.org/10.1097/00002030-200003100-00019

34. Owens S, Harper G, Amuasi J, Offei-Larbi G, Ordi J, Brabin BJ. Placental malaria and immunity to infant measles. Arch Dis Child 2006;91:507–508. https://dx.doi.org/10.1136%2Fadc.2005.085274.

35. Addy PA, Esena RK, Atuahene SK. Possible contributing factors to the paucity of yellow fever epidemics in the Ashanti region of Ghana, West Africa. East Afr Med J. 1996;73:3-9.

36. Ondiba IM, Oyieke FA, Athinya DK, Nyamongo IK, Estambale BBA. Larval species diversity, seasonal occurrence and larval habitat preference of mosquitoes transmitting

Rift Valley fever and malaria in Baringo County, Kenya. Parasite Vector 2019;12:295. https://doi.org/10.1186/s13071-019-3557-x.

37. WHO. Malaria Fact Sheet 2020. https://www.who.int/news-room/fact-sheets/detail/malaria. Accessed 04 February 2021.

January 6, 2022

Dear Editor

PLoS One

Response to comments

We thank you for your efforts both in giving our manuscript quality review and expediting the process. As suggested and requested by the Editor and the Reviewer, we have provided responses to the concerns raised. Our responses are appended below:

Editor's comments

1. We have now adapted the manuscript as suggested to the journal's style
2. Online profile is now update to include the ORCID number of the corresponding
3. We have now removed the phrase "data not shown" and associated sentence from the manuscript since the data are not a core part of the research being presented in our study.
4. Figures 1 and 2 are original and we generated by the software QGIS (version 3.14). This was stated in the statistical analyses. So, they have no copyright issues.
5. Data availability statement has been revised and the website included. The file containing the minimal data is also included as supplementary file.

Reviewer #1: General comments

This is an important subject in Africa, the continent with the highest burden of malaria, but of recently experiencing outbreaks due to viral diseases.

Line 69: Authors should change the word "Precious" to "Previous"

Response – Thank you for the observation. It has now been corrected

Line 207. The statement is not correct. Rephrase to read "Fever in each of African Region and Africa as a whole"

Response – Thank you. This is now corrected as requested

Line 236: The word ITN has been introduced without being defined anywhere in the manuscript

Response – Thank you for the observation, it is now defined at first mention (please see line 224)

Lines 236-237: The statement must be supported by appropriate References

Response – Thank you, we have now included references

Line 276: The mention of Culex mosquito is irrelevant and confusing. The mosquito species neither transmit malaria no Yellow fever.

Response – Thank you, we have deleted Culex mosquitoes as advised
