# Supplementary material for: Malaria transmission in Africa: Its relationship with yellow fever and measles
Source: PLoS One. 2022 May 4;17(5):e0268080. doi: 10.1371/journal.pone.0268080 (PMC9067666; doi:10.1371/journal.pone.0268080)
Supplement: Attachment — Submitted filename: Response to Reviewers Comments.docx [file pone.0268080.s002.docx]

January 6, 2022

Dear Editor

PLoS One

**Response to comments**

We thank you for your efforts both in giving our manuscript quality review and expediting the process. As suggested and requested by the Editor and the Reviewer, we have provided responses to the concerns raised. Our responses are appended below:

**Editor’s comments**

1. We have now adapted the manuscript as suggested to the journal’s style

2. Online profile is now update to include the ORCID number of the corresponding

3. We have now removed the phrase “data not shown” and associated sentence from the manuscript since the data are not a core part of the research being presented in our study.

4. Figures 1 and 2 are original and we generated by the software QGIS (version 3.14). This was stated in the statistical analyses. So, they have no copyright issues.

5. Data availability statement has been revised and the website included. The file containing the minimal data is also included as supplementary file.

**Reviewer #1: General comments**
This is an important subject in Africa, the continent with the highest burden of malaria, but of recently experiencing outbreaks due to viral diseases.
Line 69: Authors should change the word “Precious” to “Previous”

**Response** – Thank you for the observation. It has now been corrected
Line 207. The statement is not correct. Rephrase to read “Fever in each of African Region and Africa as a whole”

**Response** – Thank you. This is now corrected as requested
Line 236: The word ITN has been introduced without being defined anywhere in the manuscript

**Response** – Thank you for the observation, it is now defined at first mention (please see line 224)
Lines 236-237: The statement must be supported by appropriate References

**Response** – Thank you, we have now included references
Line 276: The mention of Culex mosquito is irrelevant and confusing. The mosquito species neither transmit malaria no Yellow fever.

**Response** – Thank you, we have deleted Culex mosquitoes as advised
