# Supplementary material for: Malaria transmission in Africa: Its relationship with yellow fever and measles
Source: PLoS One. 2022 May 4;17(5):e0268080. doi: 10.1371/journal.pone.0268080 (PMC9067666; doi:10.1371/journal.pone.0268080)
Supplement: Attachment — Submitted filename: Plos One Revision2.docx [file pone.0268080.s004.docx]

**Response to reviewer’s comments**

**Abstract**

Comment - Do you think that having only few studies indicate the relevance of your study? Better to show existing gaps that assured the valuable of your study in this section?

Response – Thank you, we have modified the statement (see lines 32-33).

Comment - please Indicate data management and clearance methods if the author implemented prior to data analysis

Response – Thank you for the comment. While we did not include this because of words limit in abstract section, we instead given the detail in the materials and methods section

Comment - Please indicate the significant limit of your analysis.

Response – Thank you. This is now included (Line 39)

Comment - The author should conclude the main findings of this work before indicating the relevance of this work.

Response – Thank you, this is now revised accordingly

**Introduction**

The background section of your text is somewhat shallow. So, I suggest the author to re-write the background section of the text in detail based on the following structure if the author agreed with this structure.

1. malaria is xxx and Malaria causes X and Y cases and problems globally and /or in africa..... Cuurently, WHO and/or other stakeholders recommend XXX public health intervention measures to control and prevent or eliminate or eradicate....malaria.

2. However, the co-existence of XXX viral infection in similar geographical location with malaria

3. Then if their any available challenges due to having similar geographical distribution..may in relation to current prevention and control mechanism, may be in relation to vaccine effectiveness....may be in related to treatment failure or anyother.

Then gabs and the relevance your finding to fill such available gabs

Response – Thank you for these important observations. We have included more information as suggested to include the gaps and the justifications for the study (see the red highlights).

Comments - I am feeling there is something cracked between the previous paragraph and this one. So, I recommend the author to incorporate few transition sentences prior to define yellow fever (i;e. a sentences which act as bridge between malaria you stated in the first paragraph and yellow fever in this paragraph to keep idea flow better.

Response – Thank you, we have now rephrased for flow of idea (see Line 61)

Comment - Please add a reference for the impact of placental malaria on the infant immunity to measle.

Response – Thank you, reference now included (see ref. 15 in line 78).

Comment – ‘’Because all the studied variables are cases and rates which are regarded as count data, a Panel Poisson regression model was adopted to understudy these relationships. Where there are observed excess zero in the cases of event considered in the study, a zero-inflated Poisson (ZIP) regression that is robust to the occurrence of zero is adopted to achieve our specified objectives’’. Such statements should be incorporated in the statistical analysis sub-heading of the tesx

Response – We have now included the statement in the statistical analysis sub-section.

Materials and methods (line 167-171).

Comments - How could manage and associate the findings of malaria with yellow fever and measles in this paper since the author collected the recorded data in different time period. So, did the author consider this time period variation while the author conclude the association of malaria incident and death with yellow fever and measles?

Response: Thank you for your comments

There are two main analyses in the study. The first analysis was the determination of annual trend change for each Malaria incidence, Death due to malaria, Measles and Yellow fever. Here, changes of these four variables were evaluated over time for each country considered in the study and each dataset were treated separately for this analysis. The time horizon for malaria incidence and death due to the malaria trend equation was 2000-2018 whereas the time horizon for Measles and Yellow fever trend equation was 1980 and 2019. This decision was based on the availability of the data obtained from the secondary source mentioned in the manuscript.

The second analysis was a regression equation where the effect of both Measles and Yellow fever on each of the Malaria incidence and Death due to malaria was investigated. Two data files were used for this analysis bearing in mind that in any regression problem there must be a pair of x and y variables. The first data file had Malaria as the dependent variable while both Measles and Yellow fever were the independent variables. The time horizon for this regression was 2000-2018 since information for malaria incidence was not available for 1980-1999 and 2019. Similarly, the second data file contained Death due to malaria as the dependent variable with Measles and Yellow fever as independent variables. The time horizon for this analysis was 2000-2017

Comment - Despite you are indicating the significance and insignificance of your finding in your result and discussion sub-heading, the author failed to indicate the significance limit of your analysis. So please add here the significance limit for your statistical analysis output.

Response – Thank you for the observation. We have now included the significance limit (see line 173).

**Results**

Comment – Figures quality should be improved

Response - Thank you. We have now used the PACE website provided by Plos to help ensure that figures meet PLOS requirements and improve the quality.

**Discussion**

Comment - According to the recent World Health Organization (WHO) published report showed, malaria and related deaths have been increased after 2019. If so, How could you harmonize your conclusion based on your finding with the WHO report?

Response – Thank you for this important observation. We have tried to provide reasons for your concern in the MS as follow “It is however important to stress that the recent WHO published report showed increase in malaria cases and associated deaths after 2019 [27]. The reasons for this have been accrued to plateaued malaria interventional funding, increase in drug and insecticide resistance, and climate change which threatens to push malaria transmission to new regions [28]. COVID-19 has further disrupted progress and implementation of various malaria intervention programs [28]”. Please see lines 256-260.

Comment - Are sure does modification of mosquito biting behavior is a factor for decline of malaria? I need more justification for this statement if your answer is yes.

Response – Thank you for your comment. This is an observation from another author which we referenced. Yes, we agree with the statement as the inability of the mosquitoes to access humans for blood meal indoor due to hindrance caused by ITN could trigger a behavioural change in mosquitoes to source for blood meal outside where there is no ITN.

Comment - would you mention few socio-ecological conditions which favour for the reduction of density of mosquito? Please elaborate it by giving example?

Response – Thank you for the observation. The examples are already given in the text. Please see the sentence below.

“changes in socio-ecological conditions in many malaria-endemic areas of Africa including changes in temperature, deforestation [22], increase use of agricultural pesticides with mosquitocidal properties [23], improved house constructions [24] could result in a decreased density of mosquitoes”.

Comment - Your justification is insuffeicent? please discuss in detail the possible reasons why malaria associated deaths is high in Africa unlike other past of the coninent.

Response – Thank you for the comment. However, the factors already highlighted are the major reasons we believe to be associated with high malaria deaths in Africa.

Comment - Are you sure do the incidence of malaria and measles case showed a positive relationship based on your finding? But you indicated in line 197-198, o]a significant negative relationship existed between malaria and measles cases (P<0.05). If so, why the author failed to keep the consistence of the findings? The author should give a clear information for this contradictory conclussion? Moreover, the author expected to discuss possible reasons why this findings happen?

Response – Thank you for this very important observation. This is a mistake from our end. We have now corrected it. We have also changed the direction of the discussion based on this change (see lines 276-287).

Comment - Why the author need to discuss the incidence of malaria in association with HIV and hebatitis? Does this is the authors objective? How could you relate it?

Response – Thank you for the observation. Since there were no enough reports to discuss the association between malaria and the studied vaccine related viruses we thought discussing the relationships between malaria and other viral agents could shed some light on the relationships between malaria and measles/YFV. Since the reviewer is not satisfied with this, we have now expunged the sentences and the references.

Comment - Are you sure does the region shared 30% of malaria associated death?

Response – Thank you for the comment. Based on the reference WHO report cited, the total malaria-related deaths in West Africa Region is more than 30%. Nigeria alone accounts for 25%.
