# Supplementary material for: Malaria transmission in Africa: Its relationship with yellow fever and measles
Source: PLoS One. 2022 May 4;17(5):e0268080. doi: 10.1371/journal.pone.0268080 (PMC9067666; doi:10.1371/journal.pone.0268080)
Supplement: Attachment — Submitted filename: Response to Reviewers Comments R2.docx [file pone.0268080.s005.docx]

Why did the authors failed to specify the exact statistical output of P-value rather than indicated as < 0.05 in the table 1? Unless and until the author provides a satisfactory explanation for why the P-value was not indicated, I will be skeptical of the data analysis's reliability.

Response – Thank you for your comment. We merely supplied the level of significance (P<0.05 or P>0.05) in the previous version. However, we have now provided the exact P value as requested by the reviewer.
