# Supplementary material for: Malaria transmission in Africa: Its relationship with yellow fever and measles
Source: PLoS One. 2022 May 4;17(5):e0268080. doi: 10.1371/journal.pone.0268080 (PMC9067666; doi:10.1371/journal.pone.0268080)
Supplement: Attachment — Submitted filename: Response to Reviewers Comments R3.docx [file pone.0268080.s006.docx]

Response to reviewer

Reviewer #2: First and foremost, I'd like to thank the authors for their success in producing a scientifically soundable article while taking into account my previous comments and suggestions. Having said that, I'd like to make a suggestion for the authors in table one.

In table one, when the author made a change after considering my previous comment, the p-value was written as o.ooo, which is not a scientifically sound way of writing. To the best of my knowledge, P-value cannot be written as 0.000 as you did in table 1. Even if the statistical software produces a p value of 0.000, the exact value cannot be equals to zero. You can verify it by double-clicking on it, which will display the actual value of P. In such cases, P value should be expressed as < o.oo1. The logic behind for such expression is the observed value can never be zero due to the presence of random error, which is unavoidable and uncontrollable. Thus, I recommend the authors to express the P-value as < 0.001 in table 1 rather than simply put 0.000. However, it should only be done if and only if the author agrees with my comment.

Response – Thank you for this important comment. We agree with the reviewer on this point. So, we have now replaced P value 0.000 with <0.001 as suggested.
